# Phase 1 study of darolutamide (ODM-201): a new-generation androgen receptor antagonist, in Japanese patients with metastatic castration-resistant prostate cancer

**DOI:** 10.1007/s00280-017-3417-3

**Published:** 2017-08-11

**Authors:** Nobuaki Matsubara, Hirofumi Mukai, Ako Hosono, Mai Onomura, Masaoki Sasaki, Yoko Yajima, Kensei Hashizume, Masanobu Yasuda, Miho Uemura, Christian Zurth

**Affiliations:** 1 Division of Breast and Medical Oncology, National Cancer Center Hospital East, 6-5-1 Kashiwanoha, Kashiwa, Chiba Japan; 2 Clinical Development, Bayer Yakuhin, Osaka, Japan; 3 Clinical Sciences Japan, Bayer Yakuhin, Osaka, Japan; 4 Clinical Statistics, Bayer Yakuhin, Osaka, Japan; 5 Clinical Pharmacology Oncology, Bayer AG, Berlin, Germany

**Keywords:** ODM-201, Darolutamide, Androgen receptor antagonist, Pharmacokinetic, Phase 1, Prostate cancer

## Abstract

**Purpose:**

This trial assessed the safety, pharmacokinetics, and efficacy of darolutamide (ODM-201), a new-generation nonsteroidal androgen receptor antagonist, in Japanese patients with metastatic castration-resistant prostate cancer (mCRPC).

**Methods:**

In this open-label, nonrandomized, two-cohort, dose-escalating phase 1 study, Japanese patients with mCRPC were enrolled after a screening period. In the single-dose period (≈1 week), darolutamide was administered at 300 mg (Cohort 1) or 600 mg (Cohort 2) on day −5 (fasting state) and day −2 (fed condition). In the subsequent multiple-dose period (fed condition), patients received darolutamide 300 mg twice daily (Cohort 1) or 600 mg twice daily (Cohort 2) for 12 weeks. Primary endpoints: evaluate safety and pharmacokinetics of darolutamide.

**Results:**

Of 12 patients enrolled, 9 received darolutamide (Cohort 1, *n* = 3; Cohort 2, *n* = 6). All 9 patients experienced ≥1 treatment-emergent adverse event (TEAE; majority Grade 1/2). Incidence of drug-related TEAEs (DR-TEAEs) was 44% (all grades; *n* = 4); most common DR-TEAE was decreased appetite (22%), and 1 serious DR-TEAE (Grade 3 nausea) was observed. No Grade ≥4 DR-TEAEs or new safety signals were observed. *C*_max_ and AUC (0–*t*_last_) were dose-dependent; pharmacokinetics of each dose appeared to be linear over time. Prostate-specific antigen response was observed in 11% (1/9) of patients. Compared with fasting status, geometric mean *C*_max_ increased 2.5-fold after 300 mg and 2.8-fold after 600 mg; geometric mean AUC (0–*t*_last_) increased 2.5-fold after both doses under fed conditions.

**Conclusions:**

Darolutamide was well tolerated at the examined doses in Japanese patients with mCRPC, without differences in safety and pharmacokinetics relative to Western patients.

**Electronic supplementary material:**

The online version of this article (doi:10.1007/s00280-017-3417-3) contains supplementary material, which is available to authorized users.

## Introduction

The global incidence of prostate cancer (PC) is approximately 1.1 million new cases per year, which accounts for 15% of all cancer cases in men [1], including Japanese men [2]. Based on the most recent estimates for Japan, there were 73,145 PC diagnoses in 2012 (incidence rate, 117.0 per 100,000) and 11,507 PC-related deaths in 2014 (mortality rate, 18.9 per 100,000) [3]. In Japan, PC is among the most common cancer types in men and the sixth highest cause of cancer-related death [2, 4].

Initially, PC is an androgen-dependent disease and will respond to androgen deprivation therapy (ADT); however, almost all patients become resistant to ADT over time and develop castration-resistant PC [5], defined as increasing prostate-specific antigen (PSA) levels despite castrate levels of testosterone or the progression of preexisting disease with or without metastases. Patients who have PC that has progressed to advanced metastatic disease with castration resistance have a poor prognosis, with median survival times historically in the range of 1–2 years, although longer durations extending to ~3 years have been reported in recent clinical trials [6–9].

Androgen receptor (AR) antagonists are nonsteroidal antiandrogen agents that bind to ARs and inhibit the androgen-induced activation of these receptors, which ultimately inhibits tumor growth and proliferation. These agents have demonstrated efficacy in patients with metastatic castration-resistant PC (mCRPC) [10–12].

Darolutamide (formerly ODM-201) is a new-generation nonsteroidal AR antagonist with a unique molecular structure. It comprises a mixture of two diastereomers, (*S*,*R*)-darolutamide (ORM-16497) and (*S*,*S*)-darolutamide (ORM-16555), which interconvert via the major metabolite keto-darolutamide (ORM-15341) preferentially to (*S*,*S*)-darolutamide; all three compounds show similar pharmacologic activity [13–15]. In preclinical trials, darolutamide demonstrated higher binding affinity compared with other AR antagonists (such as bicalutamide and enzalutamide), an antiproliferative effect and tumor growth inhibition in AR-overexpressing cells, and activity against AR mutants linked to drug resistance. In addition, darolutamide is different from other new-generation nonsteroidal AR antagonists with respect to its negligible blood–brain barrier penetration [14–16]. In early phase clinical trials with Western mCRPC patients, darolutamide has shown a good safety profile and significant reductions in PSA levels [13, 17–19].

The aim of this phase 1 trial was to assess the safety and tolerability, pharmacokinetics (PK), and antitumor activity of darolutamide in Japanese patients with mCRPC (ClinicalTrials.gov identifier: NCT02363855).

## Methods

### Trial design

**Fig. 1:**
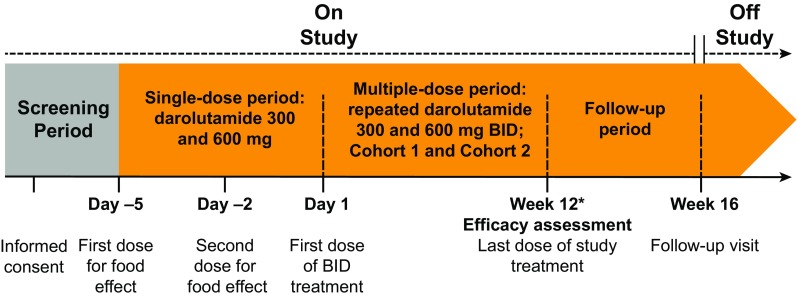
Study design. If the investigator judged that treatment with darolutamide could be continued on the current dose after the multiple-dose periods, then patients could receive darolutamide until they met one of the criteria for withdrawal. *BID* twice daily

This was an open-label, nonrandomized, two-cohort, dose-escalating phase 1 study. After a screening period (up to 4 weeks), patients entered a single-dose period (~1 week), followed by a multiple-dose period (12 weeks) and a follow-up period (4 weeks) (Fig. 1). The primary endpoints were to determine the maximum tolerated dose and evaluate dose-limiting toxicities, which were assessed by the number and intensity of all treatment-emergent adverse events (TEAEs) of darolutamide in Japanese patients with mCRPC. A secondary endpoint was to determine the PK parameters of darolutamide, diastereomers, (*S*,*R*)-darolutamide and (*S*,*S*)-darolutamide, and the major metabolite keto-darolutamide. Additional endpoints were to assess the efficacy and pharmacodynamics of darolutamide and explore the effect of food on PK.

In the single-dose period, darolutamide was administered in the fasting state on day −5 and after a usual Japanese breakfast (fed condition) on day −2 as two 150-mg tablets (300-mg dose; Cohort 1) or as four 150-mg tablets (600-mg dose; Cohort 2). Within 2 days of the single-dose period, patients were moved to the multiple-dose period, which consisted of two dose levels. The starting dose was 300 mg (Cohort 1) followed by 600 mg (Cohort 2). Cohort 2 initiated therapy based on the clinical safety evaluation after the last patient in Cohort 1 completed the 28-day period of the multiple-dose period. Patients could be added to Cohort 1 to evaluate safety and tolerability as needed. In the multiple-dose period, Cohort 1 received 300 mg twice daily (BID; total daily dose, 600 mg) and Cohort 2 received 600 mg BID (total daily dose, 1200 mg) on days 1–28. Darolutamide was given orally BID with breakfast and dinner. Treatment continued on the current dose until disease progression, intolerable toxicities, or consent withdrawal. A follow-up examination was conducted approximately 4 weeks after the end of study treatment.

### Patients

Japanese patients aged ≥20 years were eligible if they had mCRPC, defined as ongoing ADT with a luteinizing hormone-releasing hormone analogue or antagonist, or bilateral orchiectomy, and castrate level of serum testosterone [<1.7 nmol/L (50 ng/dL)] at screening and radiographic progressive disease or PSA increase of three consecutive rises, at least 1 week apart and PSA >2 ng/mL at screening; Eastern Cooperative Oncology Group performance status (ECOG PS) of 0–1; and prior treatment history with an antiandrogen for mCRPC. Patients with and without previous docetaxel-based chemotherapy were eligible for study entry. Patients were excluded if they had known brain metastases; any prior treatment for mCRPC within 4 weeks before study drug administration; use of bicalutamide within 6 weeks before the first dose of darolutamide; use of systemic corticosteroid with dose greater than the equivalent of 10 mg/day prednisone within 4 weeks before the first dose of darolutamide; or initiation of bisphosphonate or denosumab therapy within 4 weeks before the first drug administration.

### Ethics

All patients gave written informed consent for participation in the study. The study was approved by the study site’s institutional review board (National Cancer Center Institutional Review Board, Japan) and conducted in accordance with the principles of the Declaration of Helsinki and the International Council for Harmonisation guideline E6: Good Clinical Practice and applicable regulatory requirements.

### Pharmacokinetic assessments

Blood samples were taken predose and at 0.5, 1, 1.5, 3, 5, 8, 12, 24, 36, and 48-h postdose on day-5 and day-2 in the single-dose period, and predose and at 0.5, 1, 1.5, 3, 5, 8, and 12-h postdose on day 7 in the multiple-dose period. Plasma concentrations of diastereomers (*S*,*R*)-darolutamide and (*S*,*S*)-darolutamide and the major metabolite keto-darolutamide were determined using a validated high-performance liquid chromatography with tandem mass spectrometric detection (LC–MS/MS) method. PK parameters were calculated using WinNonlin software (version 5.3; Pharsight Corporation, Mountain View, CA, USA) in conjunction with the Automation Extension (Bayer AG).

### Antitumor efficacy assessments

Antitumor efficacy was assessed by PSA response, which is defined as percentage change of PSA at week 12 from baseline; tumor response was defined in accordance with the recommendations of the Prostate Cancer Clinical Trials Working Group 2 (PCWG2) [20] and Response Evaluation Criteria in Solid Tumors (RECIST) 1.1 [21]. Blood samples for PSA levels were collected at screening, once during the single-dose period (predose on day −5 or day −6; mean defined the baseline value), at three time points during the multiple-dose period (weeks 4, 8, and 12), at the end of treatment, and at the follow-up. For patients with a PSA decline from baseline at week 12, PSA progression was defined as the date of documented PSA increase ≥25% and absolute increase ≥2 ng/mL above the nadir, which was to be confirmed by a second value obtained ≥3 weeks later. For patients without a PSA decline from baseline at week 12, PSA progression was defined as the date of documented PSA increase ≥25% along with an absolute increase from baseline ≥2 ng/mL, which was to be confirmed by a second value obtained ≥3 weeks later.

Radiologic assessment [magnetic resonance imaging/computed tomography (CT)] of metastatic soft-tissue lesions was performed on all suspected sites of disease and evaluated locally at the study site using RECIST 1.1 criteria. Soft-tissue lesions were imaged using CT scans at screening and within 1 week before the last visit in the multiple-dose period. RECIST 1.1 criteria were used for the evaluation of disease progression. Bone metastases were assessed by radionuclide bone scintigraphy (bone scans) with ^99m^Technetium (^99m^Tc) at screening and within 1 week before the last visit in the multiple-dose period.

### Safety and tolerability

Safety evaluations were performed until week 12 and included results of physical examinations, 12-lead electrocardiogram (ECG), Holter ECG, vital signs (blood pressure, pulse rate, and body temperature), body weight, adverse events (AEs), and laboratory examinations. All AEs were graded using the National Cancer Institute Common Terminology Criteria for Adverse Events, version 4.03 (NCI CTCAE v4.03). Laboratory examinations were performed at screening; predose on day −5 or day −6, day −2, and day 1; at day 7 before breakfast; at each subsequent visit during the multiple-dose period; at the end of treatment; and at the follow-up/discontinuation.

### Statistical analysis

The safety analysis set was defined as all patients who received ≥1 dose of darolutamide, the full analysis set was defined as all patients who were assigned to study treatment, and the PK analysis set was defined as all patients who had valid PK data. No formal statistical sample size estimation was performed, because this phase 1 trial is standard phase 1, 3 + 3 design of toxicity assessment.

Statistical evaluation was performed using SAS release 9.2 or higher (SAS Institute Inc., Cary, NC, USA). All variables were analyzed by descriptive statistics. For PK parameters, arithmetic mean, SD and coefficient of variation (CV), geometric mean (GM), geometric SD (re-transformed SD of the logarithms) and CV; minimum, median, maximum values; and the number of measurements were calculated for darolutamide, diastereomers (*S*,*R*)-darolutamide and (*S*,*S*)-darolutamide, and major metabolite keto-darolutamide. Individual and GM concentration versus time curves were plotted by cohort using both linear and semilogarithmic scales. PK parameters of time to maximum observed drug concentration (*t*_max_) and time of the last data point greater than the lower limit of quantitation (*t*_last_) were described using minimum, maximum, and median as well as frequency counts. For maximum observed drug concentration (*C*_max_) and area under the concentration versus time curve (AUC), 90% confidence interval for the GM ratio (fed/fast) was calculated.

## Results

### Patient disposition and demographics

**Fig. 2:**
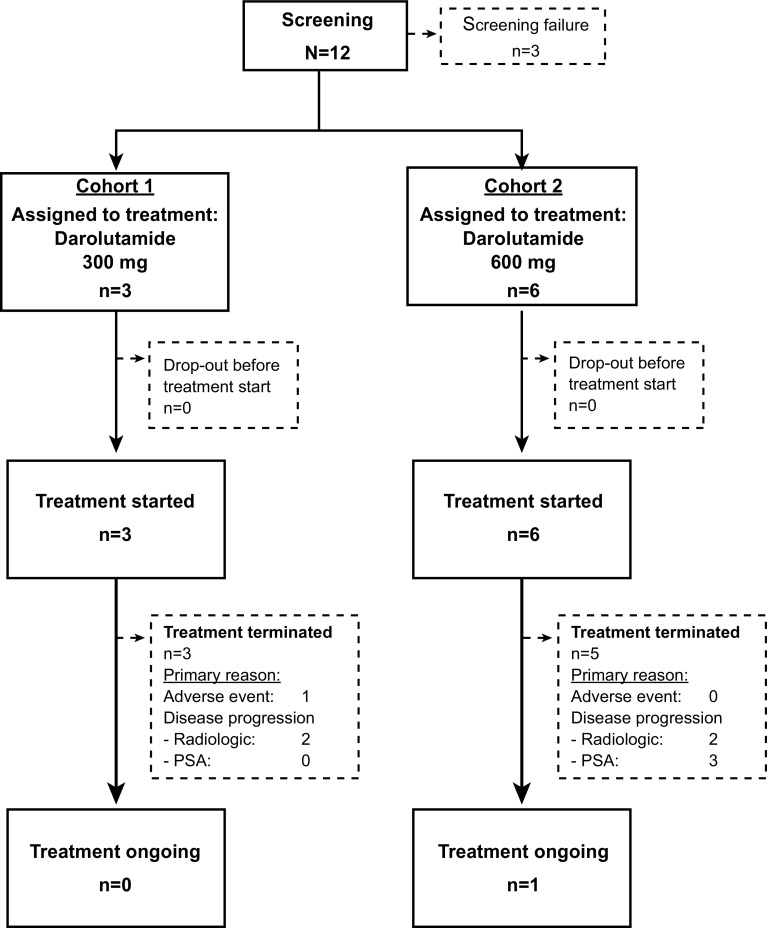
Patient disposition (all patients, cut-off date: May 9, 2016)

Patient disposition is shown in Fig. 2. Of the 12 patients enrolled, nine were assigned to receive darolutamide: three patients received darolutamide 300 mg (Cohort 1), and six received darolutamide 600 mg (Cohort 2). All nine patients received two single doses of darolutamide in the single-dose period and then started multiple-dose treatment on day 1 of the multiple-dose period. At the time of data cut (May 9, 2016), 8 of 9 (89%) patients had terminated multiple-dose treatment and completed the safety follow-up, and 1 patient (11%) had continued treatment with darolutamide without disease progression. Treatment discontinuation was primarily due to disease progression (four patients had radiologic progression, and three had PSA progression).

Demographic and baseline characteristics are presented in Table 1. Overall, the median (range) age of the nine male Japanese patients was 70.0 (64–83) years; median body mass index was 23.2 (range 20–28) kg/m^2^, and median PSA level was 39 (range 5–290) µg/L. Mean Gleason score was 8. All patients had an ECOG PS of 0–1. All nine patients had received prior hormonal therapy for mCRPC; four patients received chemotherapy.

**Table 1:** Demographics and patient characteristics

	Cohort 1 300 mg BID *n* = 3	Cohort 2 600 mg BID *n* = 6	Total *N* = 9
Median (range) age, years	68.0 (67–73)	73.0 (64–83)	70.0 (64–83)
Mean (SD) age	69.3 (3.2)	73.7 (7.6)	72.2 (6.6)
Median (range) weight, kg	61.9 (59–69)	58.9 (53–77)	61.9 (53–77)
Median (range) height, cm	165.5 (163–170)	158.7 (152–170)	162.8 (152–170)
Median (range) body mass index, kg/m^2^	23.4 (20–25)	23.0 (21–28)	23.2 (20–28)
Median (range) PSA, µg/L	61 (39–290)	32 (5–260)	39 (5–290)
Mean (SD) PSA	130 (139)	99 (118)	109 (117)
ECOG PS, *n* (%)^a^			
0	2 (66.7)	4 (66.7)	6 (66.7)
1	1 (33.3)	2 (33.3)	3 (33.3)
EOD at screening, *n* (%)^b^			
0	1 (33.3)	0	1 (11.1)
1	0	0	0
2	1 (33.3)	2 (33.3)	3 (33.3)
3	1 (33.3)	4 (66.7)	5 (55.6)
4	0	0	0
Prior therapy, *n* (%)			
Chemotherapy/hormonal	1 (33.3)/3 (100.0)	0 (0)/6 (100.0)	1 (11.1)/9 (100.0)
Docetaxel	2 (66.7)	2 (33.3)	–
Abiraterone	2 (66.7)	3 (50.0)	–
Enzalutamide	3 (100.0)	3 (50.0)	–

*BID* twice daily, *ECOG PS* Eastern Cooperative Oncology Group performance status, *EOD* extent of disease, *PSA* prostate-specific antigen

^a^ECOG PS: 0 = fully active; 1 = restricted active; 2 = ambulatory and capable of all self-care; 3 = capable of limited self-care; 4 = completely disabled

^b^EOD (bone scan findings/evaluation of bone metastases): 0 = normal or abnormal because of benign bone disease; 1 = <6 metastatic sites; 2 = 6–20 metastatic sites; 3 = >20 lesions but not a superscan; 4 = superscan (i.e., >75% of the ribs, vertebrae, and pelvic bones)

Median percentage of patients who received the planned dose of darolutamide was 99.2% (range 98–100%) for the 300-mg BID dose and 99.6% (range 73–100%) for darolutamide 600-mg BID dose. Mean percentage of patients who received the planned dose of darolutamide was slightly higher in the 300-mg BID dose cohort (99.2%) versus the 600-mg BID dose cohort (95.2%), because 1 patient in the latter dose cohort had a lower compliance of 73%. Mean (SD) actual daily dose of darolutamide was 595.1 (4.8) mg in Cohort 1 and 1142.9 (129.5) mg in Cohort 2.

### Pharmacokinetic assessment

#### Darolutamide (mixed diastereomers)

##### Single-dose period

Overall, median *t*_max_ was 3–6 h for darolutamide, demonstrating slow absorption; terminal half-life was in the range of 10–15 h. The PK parameters for darolutamide in the single-dose period under fasted and fed conditions are shown in Table 2. *C*_max_ and AUC (0–*t*_last_) values were higher with 600 versus 300 mg under fasting and fed conditions. The CV for AUC (0–*t*_last_) was higher for 300 mg (69.6%) versus 600 mg (41.4%) and higher than those under fed conditions (20.1 and 24.0%, respectively). Darolutamide achieved peak concentrations between 3 and 5 h postdose in the fasted state and between 3 and 8 h postdose in the fed state. In addition, *t*_max_ was observed later under fed versus fasting conditions (Supplemental Fig S1).

**Table 2:** Summary of darolutamide pharmacokinetic parameters for the single-dose period (fasting and fed conditions)

Parameter	Dose (mg)	*n*	Day −5 (fasting)	Day −2 (fed)
			Geom mean (CV%)	Geom mean (CV%)
AUC, μg· h/mL	300	2	24.2 (36.7)	45.5 (23.7)
	600	4	19.0 (34.8)	63.5 (28.9)
AUC(0–*t*_last_), μg· h/mL	300	3	15.7 (69.6)	39.0 (20.1)
	600	6	22.0 (41.4)	55.6 (24.0)
AUC(0–*t*_last_)/*D*, h/L	300	3	0.052 (69.6)	0.130 (20.1)
	600	6	0.037 (41.4)	0.093 (24.0)
AUC/D, h/L	300	2	0.081 (36.7)	0.152 (23.7)
	600	4	0.032 (34.8)	0.106 (28.9)
AUC(0–12), μg· h/mL	300	3	8.1 (84.8)	20.4 (15.3)
	600	6	10.8 (38.9)	25.1 (15.3)
AUC(0–12)/*D*, h/L	300	3	0.027 (84.8)	0.068 (15.3)
	600	6	0.018 (38.9)	0.042 (15.3)
*C*_max_, μg/mL	300	3	1.05 (92.9)	2.59 (7.57)
	600	6	1.26 (41.3)	3.50 (12.1)
*C*_max_/*D*, 1/L	300	3	0.004 (92.9)	0.009 (7.57)
	600	6	0.002 (41.3)	0.006 (12.1)
t_1/2_, h	300	2	15.2 (2.84)	14.8 (16.4)
	600	4	10.1 (21.2)	14.1 (36.7)
*t*_max_, h	300	3	3.05^a^ (2.95–4.97^b^)	4.92^a^ (2.98–8.00^b^)
	600	6	4.85^a^ (3.05–4.92^b^)	6.29^a^ (4.93–7.90^b^)

*AUC* area under the concentration versus time curve, *AUC(0–t*_*last*_ *)* AUC from time 0 to time of last data point, *C*_*max*_ maximum observed drug concentration, *CV* *%* geometric coefficient of variation, *D* dose-normalized, *t*_*1/2*_ half-life, *t*_*max*_ time to reach *C*_max_

^a^Median

^b^Range

Under both fasted and fed conditions, dose-normalized values for *C*_max_ (*C*_max_/D), AUC (AUC/D), and AUC(0–*t*_last_) (AUC[0–*t*_last_]/D) showed no relevant differences between the 300- and 600-mg doses, although AUC/D and *C*_max_/*D* tended to be lower for darolutamide 600 mg.

Administration of darolutamide as a single oral dose under fed conditions demonstrated that bioavailability of darolutamide was 2.5- and 2.8-fold higher (after 300 and 600 mg, respectively) versus darolutamide given in fasting conditions. Similarly, the AUC(0–*t*_last_) of darolutamide for the fed state was 2.5-fold higher after 300 and 600 mg compared with the fasting state (Supplemental Table S1).

##### Multiple-dose period

Darolutamide demonstrated a relatively flat PK profile at steady state that was most likely associated with the short dosing interval and its terminal half-life (Supplemental Fig S1). The PK parameters for darolutamide in the multiple-dose period are shown in Table 3. On day 7 of the multiple-dose (md) period, darolutamide *C*_max_ was reached 3–11 h after the dose taken with breakfast, with median *t*_max,md_ values of 4.98 and 5.48 h for 300 mg BID and 600 mg BID, respectively. Geometric mean *C*_max,md_ values for darolutamide on day 7 were 4.60 and 5.80 μg/mL for 300 mg BID and 600 mg BID, respectively, which is approximately 1.8 and 1.7 times higher versus *C*_max_ values achieved after 300- and 600-mg single doses under fed conditions (2.59 and 3.50 μg/mL). Geometric mean AUC values for AUC_tau_ (0–12)_md_ were 44.4 and 58.7 μg h/mL for darolutamide 300 mg BID and 600 mg BID, corresponding to a 1.3-fold increase in exposure after multiple dosing with 600 mg BID versus 300 mg BID. Mean linearity factor (R_LIN_) was comparable between the doses (0.910 for 300 mg BID, 0.961 for 600 mg BID). The dose-normalized parameter *C*_max_/*D*_md_ and AUC_tau_(0–12)/*D*_md_ does not indicate any relevant differences between the 2 dose levels.

**Table 3:** Summary of darolutamide pharmacokinetic parameters for multiple-dose period (day 7)

Parameter	Dose, BID (mg)	*n*	Geom mean (CV%)
AUC_tau_ (0–12),_md_, μg·h/mL	300	3	44.4 (18.2)
	600	6	58.7 (26.9)
AUC_tau_ (0–12)/D_md_, h/L	300	3	0.148 (18.2)
	600	6	0.098 (26.9)
*C*_max,md_, μg/mL	300	3	4.60 (10.3)
	600	6	5.80 (22.0)
*C*_max_/*D*_md_, 1/L	300	3	0.0153 (10.3)
	600	6	0.0097 (22.0)
*R*_A_AUC	300	3	2.18 (26.0)
	600	6	2.34 (27.8)
*R*_A_ *C*_max_	300	3	1.78 (17.8)
	600	6	1.66 (24.6)
*R*_LIN_	300	2	0.910 (4.56)
	600	4	0.961 (13.9)
*t*_max,md_, h^a^	300	3	4.98 (3.00–8.10)
	600	6	5.48 (2.87–10.9)

*AUC* area under the concentration versus time curve, *C*_*max*_ maximum observed drug concentration, *CV* geometric coefficient of variation, *D* dose-normalized, *md* multiple dose, *R*_*A*_ accumulation ratio, *R*_*LIN*_ mean linearity factor, *t*_*max*_ time to reach *C*_max_

^a^Median (range)

##### Darolutamide diastereomers (*S*,*R*)-darolutamide and (*S*,*S*)-darolutamide

Median *t*_max_ was shorter for diastereomer (*S*,*R*)-darolutamide versus diastereomer (*S*,*S*)-darolutamide at both darolutamide dose levels when administered as single or multiple doses (Supplemental Tables S2 for single dose and S3 for multiple doses). Exposure to diastereomer (*S*,*R*)-darolutamide was less versus diastereomer (*S*,*S*)-darolutamide. The ratio of diastereomer (*S*,*R*)-darolutamide AUC(0–*t*_last_) to diastereomer (*S*,*S*)-darolutamide was approximately 1:4 (fasting) and 1:5 (fed) after a single dose of 300 mg, and approximately 1:7 (fasting) and 1:8 (fed) after a single dose of 600 mg.

##### Metabolite keto-darolutamide

The *C*_max_ of major metabolite keto-darolutamide was higher compared with darolutamide at both the 300- and 600-mg dose levels when administered as either single or multiple doses (Supplemental Tables S2, S3). Exposure to metabolite keto-darolutamide was 1.28-fold (fasting) and 1.33-fold (fed) higher compared with darolutamide after a single dose of 300 mg, and 1.44-fold (fasting) and 1.61-fold (fed) higher after a single dose of 600 mg. A similar food effect was observed for *C*_max_. Food had no effect on *t*_max_ (Supplemental Tables S2, S3).

### Safety

All 9 (100%) patients reported ≥1 TEAE. Drug-related TEAEs were reported for 4 of 9 (44%) patients: 2 patients each in Cohorts 1 and 2. The 7 drug-related TEAEs reported in the study were Grade 3 nausea, Grade 2 vomiting, Grade 1 headache, Grade 1 decreased appetite (all 4 events reported for the same patient in Cohort 1), an additional case of Grade 1 decreased appetite (1 patient in Cohort 1), Grade 2 amylase increased (1 patient in Cohort 2), and Grade 1 pyrexia (1 patient in Cohort 2). Drug-related TEAEs of Grades 4 or 5 were not observed. Serious TEAEs were reported for 3 of 9 (33%) patients: Grade 3 nausea (1 patient; 300 mg BID), Grade 2 enterocolitis (1 patient; 300 mg BID), and Grade 2 malaise (1 patient; 600 mg BID). Only nausea was considered to be drug related. The frequency or severity of drug-related TEAEs and serious TEAEs did not increase with darolutamide 600 mg BID versus 300 mg BID.

There were no clinically relevant changes in vital signs or ECG. All laboratory evaluations were considered unrelated to study drug except for 1 patient who had an increase in amylase (Grade 2) treated with darolutamide 600 mg BID. Darolutamide had no drug effect on serum concentrations of follicle-stimulating hormone (FSH), luteinizing hormone (LH), testosterone, or dihydrotestosterone (DHT).

### Efficacy

#### PSA kinetics

In Cohort 1, mean PSA increased from 132.0 µg/L (*n* = 3) at baseline to 276.6 µg/L at week 8 (*n* = 2) and then was 93.1 µg/L at week 12 (*n* = 1), which corresponded to a mean (SD) change of 84.8% (14.6%; median change, 81.3%) from baseline to week 12 (Supplemental Tables S4, S5). No PSA response was observed in Cohort 1. In Cohort 2, mean PSA increased from 97.0 µg/L (*n* = 6) at baseline to 178.1 µg/L at week 12 (*n* = 5), which corresponded to a mean (SD) change of 55.2% (95.7%; median change, 53.0%; Supplemental Tables S4, S5). Changes in PSA demonstrated high interpatient variability as evidenced by a change from baseline to week 12 in PSA concentration that varied from +72.3 to +100.9% in Cohort 1, and from −84.6 to +211.1% in Cohort 2 (Supplemental Table S5). Similarly, assessment of changes from baseline at any time during or after treatment demonstrated maximum decreases in PSA of 0.7% (Cohort 1) and 84.6% (Cohort 2) and maximum increases of 100.9% (Cohort 1) and 72.2% (Cohort 2; Supplemental Table S5). Only 1 of 9 patients showed a PSA response (PSA decline ≥50% from baseline) at week 12 (600 mg BID cohort).

#### RECIST and bone scan response

At time of screening, six patients (67%) had only bone metastases, 1 (11%) had only visceral metastases, and 2 (22%) had both bone and visceral metastases. None of the three patients evaluable for the assessment of soft-tissue involvement achieved complete response or partial response, one had stable disease, and two experienced progressive disease (Supplemental Table S6). None of the six patients who had only bone metastases achieved a complete or partial response.

The extent-of-disease (EOD) grades shown by ^99m^Tc bone scintigraphy performed at screening (baseline), visit 8 (week 12), and visit 14 (month 9) are reported in Supplemental Table S7. Bone scan results showed no progression of bone lesions in this study.

## Discussion

This dose-escalating phase 1 study was the first clinical study to evaluate safety and PK of darolutamide in Japanese patients with mCRPC. Darolutamide administered as a single 300- or 600-mg once-daily dose (with and without food) or as multiple doses of 300 mg BID or 600 mg BID for a median treatment duration of 84 days (range 31–328) was well tolerated in this heavily treated population, and overall toxicities were consistent with the known safety profile of darolutamide in a previously reported phase 1 trial in a Western study population [13, 17].

Our results show that there are no remarkable differences in PK parameters between Japanese and Western patients with mCRPC [13, 17]. For example, Western patients in the ARAFOR study who were administered a single dose of darolutamide 600 mg had *C*_max_ and AUC_0–48_ values approximately twofold greater in the fed versus fasted state compared with 2.8- and 2.5-fold greater in Japanese patients, and fed-state *t*_max_ values of 4.0 versus 6.3 h, respectively [17]. Similar PK results were also observed in the ARADES study in which Western patients received a daily dose of 200–1800 mg of darolutamide. On day 1, median *t*_max_ values were 3.0–5.1 and 1.5–5.0 h for darolutamide and keto-darolutamide, respectively. At steady state, mean half-life of darolutamide was 15.8 h, independent of dose, and 10.0 h for keto-darolutamide [13]. Thus, there is no need for dose adjustment of darolutamide based on Japanese ethnicity. Similar to the Western patient studies, a significant food effect was observed on the bioavailability of darolutamide in that absorption was slower in the fasted condition, and AUC and *C*_max_ were increased twofold along with a prolongation of *t*_max_ under fed conditions. As the current Japanese PK data support the PK findings observed in the previous Western patient population studies, these collective absorption and exposure data suggest that darolutamide should be taken with food. Finally, *C*_max_ and AUC(0–*t*_last_) of darolutamide increased by dose, while the accumulation ratios for darolutamide calculated from *C*_max_ (R_A_ *C*_max_) and AUC (R_A_AUC), as well as the *R*_LIN_ of PK after repeated administration of 300 and 600 mg, were comparable for the two tested doses, suggesting that the PK of each dose was linear over time.

All patients in this study had received prior systemic treatment for mCRPC, which likely affected the observed efficacy of darolutamide. A complete response or partial response was not reported at either dose level; however, seven patients had a history of extensive anticancer treatment that included new AR antagonist agents (abiraterone and enzalutamide) and/or chemotherapy (docetaxel and cabazitaxel). Similarly, only one patient achieved a PSA response (patient had a PSA decline ≥50% from baseline at week 12), which is not unexpected, considering that all patients had received previous therapy for mCRPC.

Similar to previous early phase clinical studies in mainly Western patients, most AEs were Grade 1–2 [13, 17], and drug-related TEAEs included vomiting, headache, decreased appetite, increased amylase, and pyrexia. Only 1 serious TEAE (Grade 3 nausea) was considered drug related. All laboratory toxicities were considered unrelated to darolutamide treatment except for amylase increase in one patient (600 mg BID). ECG findings were not clinically significant, and darolutamide had no observed effect on FSH, LH, testosterone, or DHT concentrations. Overall, a dose-dependent increase in the frequency or severity of AEs was not observed. Our results confirmed that darolutamide has a favorable toxicity profile in Japanese mCRPC patients.

A limitation of the study is that any prior systemic anticancer therapy was allowed, confounding evaluation of efficacy in this heavily pretreated patient population; it is not known whether the main objectives of the study (i.e., safety and PK assessments) were affected by pretreatment. In addition, with only three and six patients having received darolutamide 300 and 600 mg, respectively, the sample sizes were too small to reliably demonstrate dose proportionality.

## Conclusions

Darolutamide was well tolerated in heavily treated mCRPC Japanese patients up to 600 mg BID, and no new safety signals were observed. There was no remarkable difference in PK between Japanese and Western patients. Exposures of darolutamide were increased with dose, with the PK of each dose appearing to be linear over time. Similar to the relative bioavailability observations in Western patient studies, darolutamide administered under fed conditions resulted in delayed absorption of the drug, with an increase in *C*_max_ and AUC compared with fasted conditions.

## Electronic supplementary material

Below is the link to the electronic supplementary material.

Supplementary material 1 (EPS 627 kb)

Supplementary material 2 (DOCX 41 kb)
