# Supplementary material for: Phase 1 study of darolutamide (ODM-201): a new-generation androgen receptor antagonist, in Japanese patients with metastatic castration-resistant prostate cancer
Source: Cancer Chemother Pharmacol. 2017 Aug 11;80(6):1063–72. doi: 10.1007/s00280-017-3417-3 (PMC5686265; doi:10.1007/s00280-017-3417-3)
Supplement: Supplementary file 2 — Supplementary material 2 (DOCX 41 kb) [file 280_2017_3417_MOESM2_ESM.docx]

# Supplemental Tables

**Table S1**. Food Effect on the Pharmacokinetics of Darolutamide and Its Metabolite ORM-15341 (Pharmacokinetic Analysis Set)

| **Dose** | **Parameter** | **Ratio Fed/Fast [90% CI]** |
| --- | --- | --- |
| **Darolutamide** | | |
| 300 mg (n=3) | C_max_ | 2.46 (0.62–9.79) |
|  | AUC(0–t_last_) | 2.49 (0.63–9.89) |
| 600 mg (n=6) | C_max_ | 2.78 (2.00–3.88) |
|  | AUC(0–t_last_) | 2.53 (1.99–3.21) |
| **(S,R)-Daralutamide** | | |
| 300 mg (n=3) | C_max_ | 2.11 (0.64–6.95) |
|  | AUC(0–t_last_) | 2.02 (0.56–7.30) |
| 600 mg (n=6) | C_max_ | 2.56 (1.81–3.62) |
|  | AUC(0–t_last_) | 2.18 (1.76–2.70) |
| **(S,S)-Daralutamide** | | |
| 300 mg (n=3) | C_max_ | 2.78 (0.68–11.4) |
|  | AUC(0–t_last_) | 2.60 (0.65–10.5) |
| 600 mg (n=6) | C_max_ | 2.74 (1.98–3.80) |
|  | AUC(0–t_last_) | 2.57 (2.02–3.28) |
| **Keto-Daralutamide** | | |
| 300 mg (n=3) | C_max_ | 2.77 (0.73–10.6) |
|  | AUC(0–t_last_) | 2.59 (0.73–9.18) |
| 600 mg (n=6) | C_max_ | 3.29 (2.41–4.49) |
|  | AUC | 2.83 (2.34–3.42) |

AUC=area under the concentration versus time curve; AUC(0–t_last_)=AUC from time 0 to last concentration >LLOQ; C_max_=maximum observed drug concentration.

**Table S2.** Summary of Diastereomers (S,R)-Daralutamide and (S,S)-Daralutamide and Major Metabolite Keto-Daralutamide Pharmacokinetic Parameters for the Single-Dose Period (Fasting and Fed Conditions)

|  | **Dose, mg** |  | **Diastereomer**  **(S,R)-Daralutamide** | |  | **Diastereomer**  **(S,S)-Daralutamide** | |  | **Major Metabolite**  **Keto-Daralutamide** | |
| --- | --- | --- | --- | --- | --- | --- | --- | --- | --- | --- |
|  |  | **N** | **Day –5 (Fasting)** | **Day –2**  **(Fed)** | **N** | **Day –5 (Fasting)** | **Day –2**  **(Fed)** | **N** | **Day –5 (Fasting)** | **Day –2**  **(Fed)** |
|  |  |  | **Geometric Mean (CV%)** | | | | | | | |
| AUC, µg·h/mL | 300 | 3 | 3.46 (59.7) | 6.49 (16.4)^b^ | 3 | 15.19 (55.7) | 38.97 (24.9)^b^ | 3 | 22.74 (49.9) | 79.44 (63.0) |
|  | 600 | 3 | 2.14 (30.1) | 6.02 (19.9)^c^ | 4 | 16.68 (36.4) | 57.35 (30.6) | 5 | 31.46 (71.4) | 96.54 (54.7) |
| AUC(0–t_last_), µg·h/mL | 300 | 3 | 3.06 (69.8) | 6.20 (13.5) | 3 | 12.57 (69.9) | 32.71 (21.7) | 3 | 19.94 (51.6) | 51.60 (85.8) |
|  | 600 | 6 | 2.66 (36.5) | 5.81 (17.6) | 6 | 19.26 (43.0) | 49.50 (25.6) | 6 | 31.55 (59.4) | 89.19 (50.0) |
| AUC(0–t_last_)/D, h/L | 300 | 3 | 0.0102 (69.8) | 0.0207 (13.5) | 3 | 0.0419 (69.9) | 0.109 (21.7) | 3 | 0.0668 (51.6) | 0.173 (85.8) |
|  | 600 | 6 | 0.00444 (36.5) | 0.00968 (17.6) | 6 | 0.0321 (43.0) | 0.0825 (25.6) | 6 | 0.0528 (59.4) | 0.149 (50.0) |
| AUC/D, h/L | 300 | 3 | 0.0115 (59.7) | 0.0216 (16.4)^b^ | 3 | 0.0506 (55.7) | 0.130 (24.9)^b^ | 3 | 0.0762 (49.9) | 0.266 (63.0) |
|  | 600 | 3 | 0.00357 (30.1) | 0.0100 (19.9)^c^ | 4 | 0.0278 (36.4) | 0.0956 (30.6) | 5 | 0.0527 (71.4) | 0.162 (54.7) |
| AUC(0–12), µg·h/mL | 300 | 3 | 2.06 (81.9) | 4.61 (14.4) | 3 | 6.00 (86.2) | 15.71 (16.7) | 3 | 10.27 (51.0) | 27.42 (87.6) |
|  | 600 | 6 | 1.99 (37.2) | 4.60 (14.9) | 6 | 8.77 (39.9) | 20.29 (16.4) | 6 | 15.69 (53.0) | 41.84 (38.9) |
| AUC(0–12)/D, h/L | 300 | 3 | 0.00688 (81.9) | 0.0154 (14.4) | 3 | 0.0200 (86.2) | 0.0523 (16.7) | 3 | 0.0344 (51.0) | 0.0919 (87.6) |
|  | 600 | 6 | 0.00332 (37.2) | 0.00766 (14.9) | 6 | 0.0146 (39.9) | 0.0338 (16.5) | 6 | 0.0263 (53.0) | 0.0701 (38.9) |
| CL/F, L/h | 300 | 3 | 86.8 (59.7) | 46.2 (16.4)^b^ | 3 | 19.7 (55.7) | 7.70 (24.9)^b^ | 3 | 13.1 (49.9) | 3.76 (63.0) |
|  | 600 | 3 | 280 (30.1) | 99.7 (19.9)^c^ | 4 | 36.0 (36.4) | 10.5 (30.6) | 5 | 19.0 (71.4) | 6.18 (54.7) |
| C_max,_ µg/mL | 300 | 3 | 0.38 (73.0) | 0.79 (9.09) | 3 | 0.72 (89.3) | 2.01 (9.21) | 3 | 1.29 (36.5) | 3.58 (79.7) |
|  | 600 | 6 | 0.34 (44.7) | 0.86 (17.4) | 6 | 1.03 (41.4) | 2.81 (16.4) | 6 | 1.78 (59.2) | 5.84 (31.8) |
| C_max_/D, /L | 300 | 3 | 0.00125 (73.0) | 0.00264 (9.09) | 3 | 0.00241 (89.3) | 0.00669 (9.21) | 3 | 0.00433 (36.5) | 0.0120 (79.7) |
|  | 600 | 6 | 0.000558 (44.7) | 0.00143 (17.4) | 6 | 0.00171 (41.4) | 0.00469 (16.4) | 6 | 0.00297 (59.2) | 0.00978 (31.8) |
| VZ/F, L | 300 | 3 | 2376 (103) | 892 (48.1)^b^ | 3 | 535 (97.0) | 166 (41.2)^b^ | 3 | 306 (58.9) | 70.6 (80.0) |
|  | 600 | 3 | 3294 (18.1) | 1516 (75.7)^c^ | 4 | 543 (22.2) | 209 (23.4) | 5 | 303 (31.3) | 111 (45.1) |
| t_1/2,_ h | 300 | 3 | 19.0 (37.4) | 13.4 (30.0)^b^ | 3 | 18.8 (32.3) | 15.0 (15.2)^b^ | 3 | 16.2 (15.8) | 13.0 (12.6) |
|  | 600 | 3 | 8.15 (22.3) | 10.5 (94.7)^c^ | 4 | 10.5 (20.1) | 13.9 (38.7) | 5 | 11.1 (38.5) | 12.4 (24.7) |
| t_max_, h | 300 | 3 | 3.05^a^ (0.433–4.97^b^) | 2.98^a^ (2.82–5.00^b^) | 3 | 4.97^a^ (4.97–5.12)^b^ | 7.95^a^ (4.97–8.00)^b^ | 3 | 4.97^a^ (2.95–5.11)^b^ | 4.92^a^ (2.98–5.00)^b^ |
|  | 600 | 6 | 1.26^a^ (0.950–4.90^b^) | 5.00^a^ (4.80–7.90^b^) | 6 | 4.89^a^ (4.77–7.80)^b^ | 7.80^a^ (5.03–12.1)^b^ | 6 | 2.88^a^ (1.47–7.80)^b^ | 6.29^a^ (4.93–7.90)^b^ |

AUC=area under the concentration versus time curve; AUC(0–t_last_)=AUC from time 0 to time of last data point; C_max_=maximum observed drug concentration; CV%=geometric coefficient of variation; D=dose-normalized; t_1/2_=half-life; t_max_=time to reach C_max_.

^a^Median (range); ^b^n=2; ^c^n=6.

**Supplemental Table S3.** Summary of Diastereomers (S,R)-Daralutamide and (S,S)-Daralutamide and Major Metabolite Keto-Daralutamide Pharmacokinetic Parameters for the Multiple-Dose Period on Day 7

|  | **Dose, mg** |  | **Diastereomer (S,R)-Daralutamide** |  | **Diastereomer (S,S)-Daralutamide** |  | **Major Metabolite Keto-Daralutamide** |
| --- | --- | --- | --- | --- | --- | --- | --- |
|  |  | **N** | **Day 7** | **N** | **Day 7** | **N** | **Day 7** |
|  |  | **Geometric Mean (CV%)** | | | | | |
| AUC_tau_(0–12),_md_ µg·h/mL | 300 | 3 | 5.60 (7.52) | 3 | 38.76 (20.0) | 3 | 61.96 (62.2) |
|  | 600 | 6 | 5.36 (18.8) | 6 | 53.09 (28.4) | 6 | 104.03 (48.8) |
| AUC_tau_(0–12)/D,_md_ h/L | 300 | 3 | 0.0187 (7.52) | 3 | 0.129 (20.0) | 3 | 0.208 (62.2) |
|  | 600 | 6 | 0.00893 (18.4) | 6 | 0.0885 (28.4) | 6 | 0.174 (48.8) |
| C_max,md_, µg/mL | 300 | 3 | 0.73 (23.7) | 3 | 3.90 (12.9) | 3 | 6.72 (54.6) |
|  | 600 | 6 | 0.87 (18.3) | 6 | 5.15 (25.7) | 6 | 11.05 (47.2) |
| C_max_/D,_md_,/L | 300 | 3 | 0.00244 (23.7) | 3 | 0.0130 (12.9) | 3 | 0.0225 (54.6) |
|  | 600 | 6 | 0.00144 (18.3) | 6 | 0.00858 (25.7) | 6 | 0.0185 (47.2) |
| R_A_AUC | 300 | 3 | 1.21 (18.0) | 3 | 2.47 (29.9) | 3 | 2.26 (28.9) |
|  | 600 | 6 | 1.17 (22.7) | 6 | 2.62 (29.4) | 6 | 2.48 (27.5) |
| R_A_C_max_ | 300 | 3 | 0.924 (29.4) | 3 | 1.94 (18.2) | 3 | 1.88 (19.6) |
|  | 600 | 6 | 1.01 (24.8) | 6 | 1.83 (31.1) | 6 | 1.89 (24.8) |
| R_LIN_ | 300 | 2 | 0.826 (15.0) | 2 | 0.923 (3.28) | 3 | 0.981 (0.4) |
|  | 600 | 6 | 0.891 (24.9) | 4 | 0.969 (13.5) | 6 | 1.08 (22.9) |
| t_max,md_, h | 300 | 3 | 4.87^a^ (3.00–4.98) | 3 | 4.98^a^ (3.00–11.0) | 3 | 4.98^a^ (3.00–8.10) |
|  | 600 | 6 | 3.94^a^ (2.87–5.05) | 6 | 7.96^a^ (2.87–10.9) | 6 | 4.89^a^ (2.87–7.93) |

AUC=area under the concentration versus time curve; C_max_=maximum observed drug concentration; CV=geometric coefficient of variation; D=dose-normalized; md=multiple dose; R_A_=accumulation ratio; R_LIN_=mean linearity factor; t_max_=time to reach C_max_.

^a^Median (range).

**Table S4.** PSA Serum Concentrations at Baseline and Selected Time Points During and After Treatment With Darolutamide

|  |  | **Darolutamide** | | |
| --- | --- | --- | --- | --- |
|  |  | **Cohort 1  300 mg BID** | **Cohort 2  600 mg BID** | **Total** |
|  |  | **n=3 (100%)** | **n=6 (100%)** | **N=9 (100%)** |
| Baseline,^a^  µg/L | n | 3 | 6 | 9 |
|  | Mean (SD) | 132.0 (116.9) | 97.0 (113.2) | 108.7 (108.3) |
|  | Median (range) | 75.5 (54.0–266.4) | 34.0 (5.0–259.7) | 54.0 (5.0–266.4) |
| Week 4 (Visit 5), µg/L | n | 3 | 6 | 9 |
|  | Mean (SD) | 219.1 (207.6) | 123.8 (164.2) | 155.6 (172.9) |
|  | Median (range) | 151.7 (53.6–452.0) | 29.5 (6.6–359.9) | 53.6 (6.6–452.0) |
| Week 8 (Visit 7), µg/L | n | 2 | 6 | 8 |
|  | Mean (SD) | 276.6 (291.9) | 136.8 (178.5) | 171.8 (197.8) |
|  | Median (range) | 276.6 (70.2–483.0) | 39.1 (4.7–409.3) | 54.9 (4.7–483.0) |
| Week 12 (Visit 8), µg/L | n | 1 | 5 | 6 |
|  | Mean (SD) | 93.1 (NA) | 178.1 (213.4) | 163.9 (194.0) |
|  | Median (range) | 93.1 (NA) | 49.4 (4.0–424.4) | 71.2 (4.0–424.4) |
| EOT visit,  µg/L | n | 3 | 3 | 6 |
|  | Mean (SD) | 289.3 (200.8) | 190.8 (210.4) | 240.0 (191.7) |
|  | Median (range) | 187.2 (160.0–520.6) | 75.3 (63.4–433.6) | 173.6 (63.4–520.6) |
| Follow-up visit,  µg/L | n | 3 | 5 | 8 |
|  | Mean (SD) | 387.8 (232.2) | 308.3 (211.3) | 338.1 (206.4) |
|  | Median (range) | 378.0 (160.6–624.7) | 399.4 (67.8–494.9) | 388.7 (67.8–624.7) |
| BID=twice daily; EOT=end of treatment; PSA=prostate-specific antigen.  ^a^The baseline PSA sample was collected at predose on Day –5 (or on Day –6). | | | | |

**Table S5.** Change From Baseline in PSA Serum Concentration

|  |  | **Darolutamide** | | |
| --- | --- | --- | --- | --- |
|  |  | **Cohort 1  300 mg BID** | **Cohort 2  600 mg BID** | **Total** |
|  |  | **n=3 (100%)** | **n=6 (100%)** | **N=9 (100%)** |
| Change from baseline at Week 12,  µg/L | n | 1 | 5 | 6 |
|  | Mean (SD) | 39.1 (NA) | 67.1 (94.2) | 62.5 (85.0) |
|  | Median (range) | 39.1 (NA) | 10.47 (–21.9; 173.9) | 24.77 (–21.9; 173.9) |
| Percentage change from baseline at any time until Week 12, LOCF^a^ | n | 3 | 6 | 9 |
|  | Mean (SD) | 84.8 (14.6) | 55.2 (95.7) | 65.1 (77.4) |
|  | Median (range) | 81.3 (72.3; 100.9) | 53.0 (–84.6; 211.1) | 72.3 (–84.6; 211.1) |
| Maximum percentage change from baseline at any time, LOCF^a^ | n | 3 | 6 | 9 |
|  | Mean (SD) | 56.6 (52.1) | 6.9 (55.6) | 23.5 (56.8) |
|  | Median (range) | 69.7 (–0.7; 100.9) | 17.3 (–84.6; 72.2) | 38.3 (–84.6; 100.9) |
| BID=twice daily; FAS=full analysis set; LOCF=last observation carried forward; PSA=prostate-specific antigen.  ^a^LOCF method was used for calculation of percentage changes from baseline in PSA based on the data of all 9 patients included in the full analysis set. | | | | |

| **Table S6.** Tumor Response Evaluation of Soft-Tissue Lesions (RECIST 1.1) | | | |
| --- | --- | --- | --- |
|  | **Darolutamide** | | |
|  | **Cohort 1  300 mg BID** | **Cohort 2  600 mg BID** | **Total** |
|  | **n=3 (100%)** | **n=6 (100%)** | **N=9 (100%)** |
| Best overall response RECIST |  |  |  |
| Complete response | 0 | 0 | 0 |
| Partial response | 0 | 0 | 0 |
| Stable disease | 1 (33.3) | 0 | 1 (11.1) |
| Non–complete response/non–partial response | 1 (33.3)^a^ | 5 (83.3)^a^ | 6 (66.7) |
| Progressive disease | 1 (33.3) | 1 (16.7) | 2 (22.2) |
| Not evaluable | 0 | 0 | 0 |
| Response rate^b^ |  |  |  |
| Yes | 0 | 0 | 0 |
| No | 3 (100.0) | 6 (100.0) | 9 (100.0) |
| Disease control rate^c^ |  |  |  |
| Yes | 1 (33.3) | 0 | 1 (11.1) |
| No | 2 (66.7) | 6 (100.0) | 8 (88.9) |
| BID=twice daily; RECIST=Response Evaluation Criteria in Solid Tumors.  ^a^No patients had measurable soft-tissue metastases at screening (only bone metastases).  ^b^Response rate: patient with complete response or partial response.  ^c^Disease control rate: patient with complete response, partial response, or stable disease. | | | |

**Table S7**. Extent of Bone Metastasis (EOD Grades) by Study Visit Revealed by ^99m^Tc Bone Scintigraphy

|  | **Visit** | **n** | **Extent of Disease, n (%)** | | | | |
| --- | --- | --- | --- | --- | --- | --- | --- |
|  |  |  |  | | | | |
|  |  |  | **0** | **1** | **2** | **3** | **4** |
| Cohort 1 300 mg BID  (n=3) | Screening | 3 | 1 (33.3) | 0 | 1 (33.3) | 1 (33.3) | 0 |
|  | Visit 8^a^ | 1 | 0 | 0 | 0 | 1 (100.0) | 0 |
|  | Visit 14^b^ | 0 | 0 | 0 | 0 | 0 | 0 |
| Cohort 2 600 mg BID  (n=6) | Screening | 6 | 0 | 0 | 2 (33.3) | 4 (66.7) | 0 |
|  | Visit 8^a^ | 5 | 0 | 0 | 2 (40.0) | 3 (60.0) | 0 |
|  | Visit 14^b^ | 1 | 0 | 1 (100.0) | 0 | 0 | 0 |
| Total  (N=9) | Screening | 9 | 1 (11.1) | 0 | 3 (33.3) | 5 (55.6) | 0 |
|  | Visit 8^a^ | 6 | 0 | 0 | 2 (33.3) | 4 (66.7) | 0 |
|  | Visit 14^b^ | 1 | 0 | 1 (100.0) | 0 | 0 | 0 |
| ^99m^Tc=^99m^Technetium; BID=twice daily; EOD=extent of disease.  ^a^Visit 8 = Week 12; ^b^Visit 14 = Month 9.  EOD 0 = Normal or abnormal because of benign bone disease.  EOD 1 = <6 metastatic sites.  EOD 2 = 6–20 metastatic sites.  EOD 3 = >20 lesions but not a superscan.  EOD 4 = Superscan. | | | | | | | |
